# *Clostridioides difficile* infection: traversing host–pathogen interactions in the gut

**DOI:** 10.1099/mic.0.001306

**Published:** 2023-02-27

**Authors:** Jeffrey K. J. Cheng, Meera Unnikrishnan

**Affiliations:** ^1^​ Division of Biomedical Sciences, Warwick Medical School, University of Warwick, Coventry, CV4 7AL, UK

**Keywords:** Clostridioides difficile infection, host-pathogen interaction, pathogenesis, virulence

## Abstract

*
C. difficile* is the primary cause for nosocomial infective diarrhoea. For a successful infection, *C. difficile* must navigate between resident gut bacteria and the harsh host environment. The perturbation of the intestinal microbiota by broad-spectrum antibiotics alters the composition and the geography of the gut microbiota, deterring colonization resistance, and enabling *C. difficile* to colonize. This review will discuss how *C. difficile* interacts with and exploits the microbiota and the host epithelium to infect and persist. We provide an overview of *C. difficile* virulence factors and their interactions with the gut to aid adhesion, cause epithelial damage and mediate persistence. Finally, we document the host responses to *C. difficile *, describing the immune cells and host pathways that are associated and triggered during *C. difficile* infection.

## Introduction

*Clostridioides difficile,* a Gram-positive, spore-forming, obligate anaerobic bacterium is the aetiological cause for *C. difficile* infection (CDI). This bacterium is ubiquitous in nature, readily isolated from soil, water, animals and importantly, nosocomial environments [1, 2]. According to UK national statistics, there were 14 269 cases reported in the UK between 2021/2022 and 462 100 cases in the United States in 2017 [3, 4]. Elderly and immunocompromised individuals undergoing long-term treatments with broad-spectrum antibiotics are usually at a higher risk for CDI [3]. Clinical manifestations of CDI can range from diarrhoea, pseudomembranous colitis (PMC) and in rare cases, toxic megacolon [5]. The costs of treating CDI is compounded by the high rates of recurrent infections; with repeated occurrences in approximately 20–30 % of patients [6].

Colonization of the gut by a pathogen is a highly complex process. *C. difficile* colonization is usually prevented by the native gut microbiota in healthy individuals and recent studies have revealed a number of mechanisms underlying microbiota-mediated resistance, which have been extensively reviewed elsewhere [7, 8]. Along with its relationship with the microbiota, the interactions of *C. difficile* with the host epithelium and the immune system play a crucial role in the pathogenesis and the outcome of CDI. This review will primarily focus on the pathogen and host factors that control infection at the gut interface.

## Pathogenesis of *C. difficile* infection

**Fig. 1.**
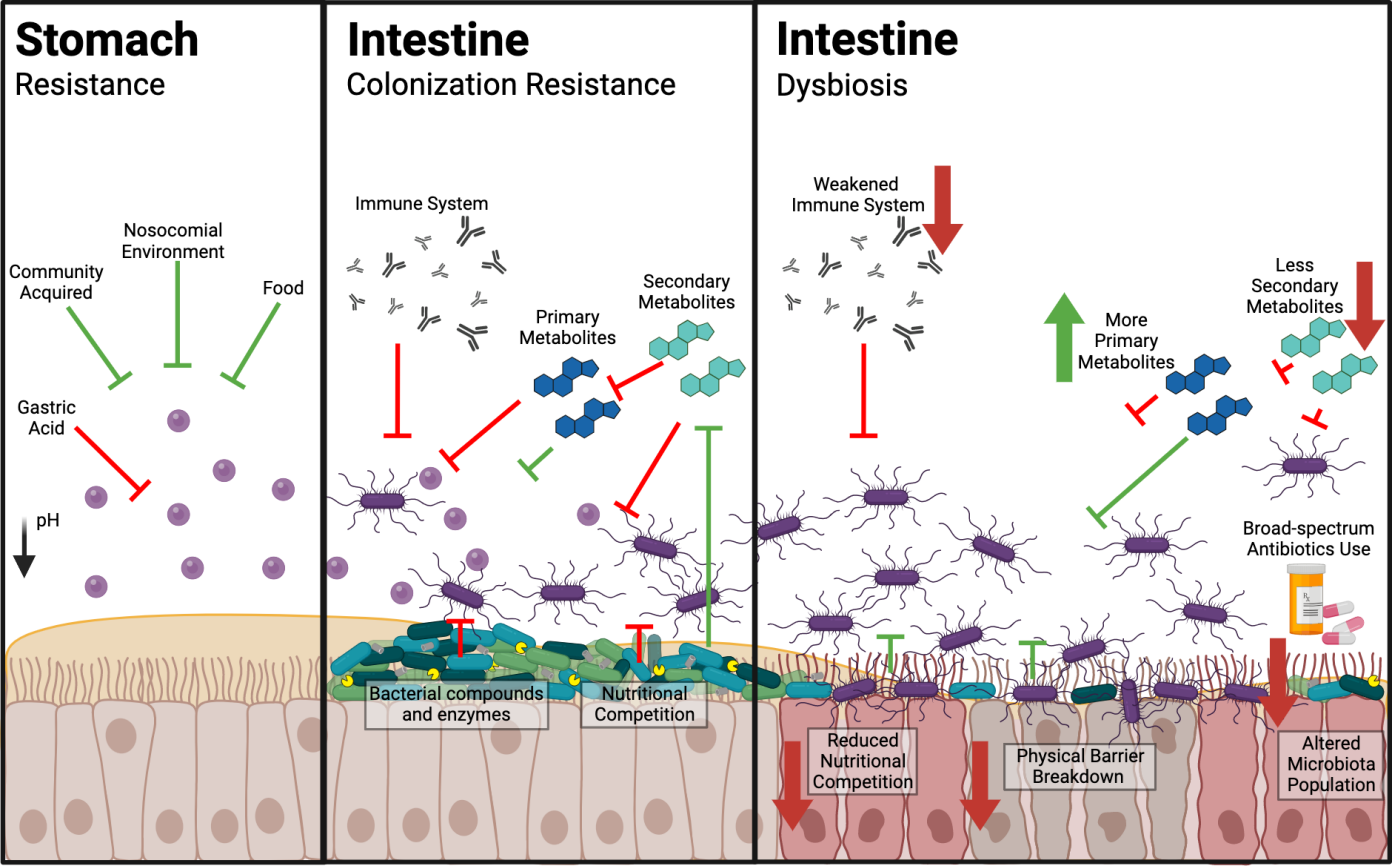
Colonization resistance and dysbiosis in the gut. Ingested spores from the environment are inhibited by stomach acid and can only germinate once it enters the intestine, with the assistance from primary metabolites such as conjugated bile salts. Secondary metabolites, promoted by the gut microbiota (e.g. deoxycholic acid and lithocholic acid) can inhibit germination. The microbiota further hinders the invading pathogen through the secretion of inhibitory compounds, mucus production, spatial and nutritional competition. The immune system can also dampen down the colonization ability and virulence during infection. *C. difficile* effectively proliferate upon the disruption of the microbiota or dysbiosis caused by broad-spectrum antibiotics. The alterations in the commensal bacteria and breakdown of the physical barrier can provide an opportunity for adhesion and colonization. Subsequent reduction in secondary metabolite conversions creates a disequilibrium for more primary bile salts, further exacerbating CDI. Additionally, an altered immune system can allow CDI to progress in severity. Created with BioRender.com.

Three main events occur during CDI: disruption of the gut microbiota, colonization and expression of virulence factors [9]. *C. difficile* forms highly resistant spores, which can survive under unfavourable environmental conditions. These spores traverse the gastrointestinal (GI) tract through ingestion, resisting physical defence mechanisms like stomach acid and mucus (Fig. 1). In the presence of co-germinating factors; predominately taurocholate (bile salts) and glycine, spores germinate into vegetative cells [10–13]. Ingested spores are also found in the gut of 1–3 % of healthy individuals [14, 15]. Treatment with broad-spectrum antibiotics results in gut dysbiosis and survival of resistant bacteria like *C. difficile* [16, 17]. When spores germinate, the vegetative cells thrive in this altered gut bacterial population. Initial bacterial colonization is mediated by a range of bacterial surface factors. *C. difficile* is believed to first attach and multiply on the mucosal surfaces before toxins are produced [18]. Ultimately, the toxins cause the restructuring of cell cytoskeleton, death of epithelial cells and leakage of fluids, leading to pseudomembranous colitis and diarrhoea (Fig. 1) [5, 19, 20].

## Role of the gut microbiota in CDI

*C. difficile* needs to overcome ‘colonization resistance’, a term used to describe the host mucosal and gut microbiota-mediated defences, to establish an infection. The host bestows physiological stresses such as luminal acidity, hypoxic mucosal micro-environments, oxidative and nitrosative stress released by immune responses [21–24]. The microbiota impedes infection through spatial limitation, nutritional competition, a supply of antagonistic enzymes, antimicrobial peptides and metabolites (Fig. 1) [8, 25–27]. Changes in population dynamics and diversity have predominately been associated with *C. difficile* susceptibility and recurrence [28–32]. In general, *Pseudomonadota* and other opportunistic pathogens were more common in CDI and CDI carriers, whilst *Bacteriodota* and were under represented [30–33]. The protective function of *Bacteriodota* and *Bacillota* have been associated with short-chain fatty acids in the fermentation of polysaccharides: acetate, butyrate, propionate and valerate [34–39], however fatty acids like succinate can be beneficial for *C. difficile* infection [40].

Secreted compounds from bacterium can inhibit foreign pathogens and alter the surrounding environment [41–44]. For example, commensal bacteria can produce peptides and bacteriocins, which can be bacteriostatic/bacteriocidal or act as signalling peptides to modulate the behaviour of other organisms [45–47]. Commensal *Bacillus thuringensis* can produce Thuricin CD, a narrow bacteriocin against *C. difficile* [48], while *Lactobacillus reuteri* competes with other commensals to produce reuterin from glycerol, in order to inhibit *C. difficile* growth [49].

Finally, the role of bile acid (BA) in the lifecycle of *C. difficile* is well described [12, 15, 50]. Intestinal BAs have been primarily described as germinants and co-germinants for *C. difficile* spores, two commonly associated BAs are cholate and chenodeoxycholate acid (CDCA). Spore germination is induced by bile acids, cholate or taurocholate, but inhibited by primary bile acid CDCA. The generation of secondary bile acids like deoxycholic acid (DCA), which is transformed from conjugated primary bile acids produced by the host, are strong inhibitors of spore germination and vegetative cell growth [51–54]. The 7α-dehydroxylation activity of the commensal *Clostridium scindens *, which results in DCA formation from cholate was associated with protection from CDI [55, 56]. Although *C. difficile* was previously thought to be a bystander, it was recently reported to deconjugate taurine-conjugated bile acids to produce cholate [57]. Additionally, the production of DCA from cholate by *C. scindens* was shown to trigger and promote *C. difficile* biofilm formation [58]. Thus, *C. difficile* interactions with the microbiota are highly complex, warranting further studies to probe the inter-species interactions occurring at the gut interface.

## *C. difficile* virulence factors

**Fig. 2.**
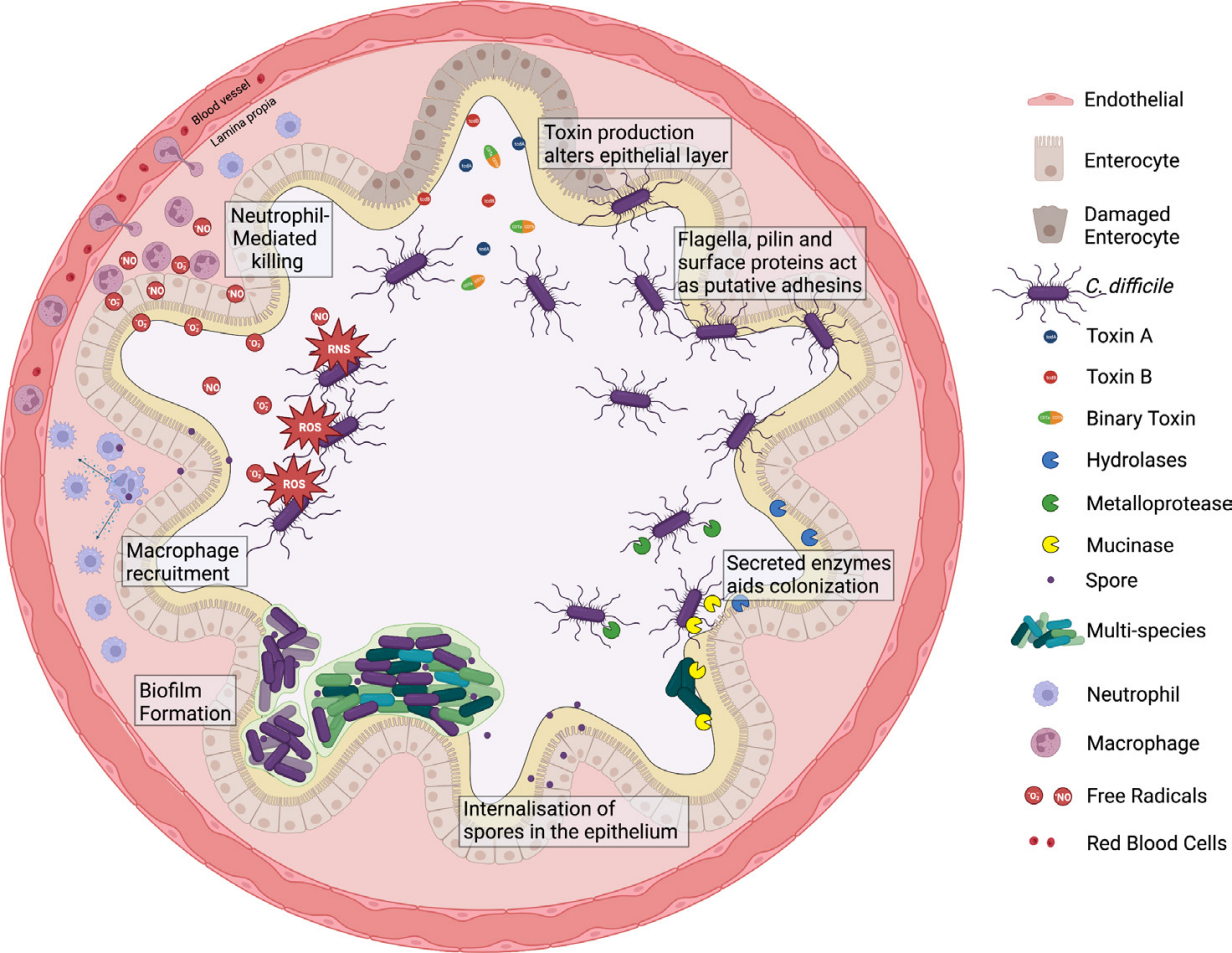
Host–pathogen interactions at the gut interface during *C. difficile* infection. Upon germination and colonization, *C. difficile* can release three toxins; A, B and binary (CDT) toxins to disrupt the epithelial surface. Remodelling of the cytoskeleton can increase adhesion, promote disease severity and degrade the protective physical barrier. Pili, flagella and surface proteins are employed by bacteria enable bacterial motility and can act as a putative adhesin to the epithelium. Furthermore, *C. difficile* can secrete several enzymes, which can activate surface proteins, degrade the mucus, or cleave off host glycoproteins. Sporulation and biofilm formation may be employed to counteract and mitigate host-derived stresses such as micro-aerophilic environment, secreted bacterial compounds and reactive oxygen/nitrogen species released by neutrophils. Neutrophil and macrophage responses are triggered in the host; these responses can be manipulated by *C. difficile* to its advantage by inducing excessive inflammation and spore internalization into macrophages (and epithelial cells). Created with BioRender.com.

*C. difficile* produces a wide range of virulence-associated factors, including many determinants that mediate bacterial attachment to the mucosal surface, that are considered key for colonization (Fig. 2). Some of these factors are discussed below.

### Toxins A and B

*C. difficile* toxins, TcdA (308 kDa) and TcdB (269.6 kDa), belong to a family of large glucosylating toxins. They are considered to be the major virulence determinants of this pathogen and have been reviewed extensively [59, 60]. Their expression is dependent on the bacterial ribotype, toxinotype and strain [61]. The genes encoding for toxin A (*tcdA*) and toxin B (*tcdB*) are found on the 19.6 kb pathogenicity locus (PaLoc) nestled between three regulatory open reading frames; *tcdR*, *tcdE* and *tcdC*. TcdR (previously known as TcdD and TexR), is a positive regulator that acts as an alternative sigma factor and mediates the recognition of the RNA polymerase core to the toxin promoters [62, 63]. TcdC is considered an anti-sigma factor that negatively regulates toxin expression by interacting with *tcdR* and/or tcdR-containing RNA polymerase [64], although there are conflicting studies on its ability to regulate toxin expression [64–71]. TcdE, a 19 kDa holin protein, is used to export TcdA and TcdB through the cell wall [72–74].

Both toxins belong in the family of large clostridial toxins (LCTs) alongside TscL of *Clostridium sordellii* [75]. The quadruple-domain structure of the toxins, which share a certain degree of homology, allows the proteins to bind to intestinal epithelial surface receptors [76] and are endocytosed in either a clathrin, caveolae-mediated or PACSIN-dynamin manner [77, 78]. Endosome acidification allows the toxins to be translocated into the cytosol [79–82] and the cleavage and activation of the enzymatic domain is catalysed by cytosolic inositol hexakisphosphate [79, 83–85]. Toxin A and B target the family of signalling G protein, called Rho GTPases (e.g. Rho, Ras and Cdc42), which acts as molecular switches for actin cytoskeleton regulation, cell movement, microtubule dynamics, vesicle trafficking, cell polarity and cell cycle progression [86, 87]. The toxins catalyse the monoglycosylation of threonine-37, leading to the abrupt stop in cell signalling and loss of cell structural integrity as a result of actin filament depolymerization, cytoskeleton perturbations and disruption of the tight cell–cell junctions [88–94]. An indirect interaction between toxin and bacterial adherence was reported in Kasendra *et al.,* as sublethal concentrations of toxin A were able to alter cell polarity of Caco-2 colonocytes, causing an increase in adhesion and penetration of the host mucosal barrier [95].

### Binary toxin

The *C. difficile* binary toxin, also known as *C. difficile* transferase (CDT) is present in approximately 4–45 % of strains isolated, but more common in hypervirulent strains such as ribotype 027 [84]. The two components of the binary toxin, CDTa and CDTb toxins are encoded on the 6.2 kb *Cdt* locus, CdtLoc, with a CDT regulator, *cdtR* upstream. Interestingly, strains can exhibit the complete CdtLoc, a 4.2 kb truncated version or be ‘absent’ with a 68 bp region [96]. Unlike LCTs, binary toxins are secreted as two separate components (classical AB toxin). The CDTb protein is responsible for receptor binding, toxin uptake, mediating pore formation and translocation of the enzymatic A component into the cytosol [97–101]. The CDTa protein is the enzymatic portion, possessing ADP-ribosyltransferase activity [102–106]. Each component is non-toxigenic on its own and toxic effects occur only when the subunits were associated with one another [107, 108].

Cell intoxication occurs with the proteolytic cleavage of the CDTb (98.8 kDa) activation domain by serine type proteases to form a mature 75 kDa protein [97, 100]. The toxin subsequently oligomerises to form heptameric pores pre- and post-receptor binding for endocytosis [109, 110]. The CDTb C-terminus binds to the extracellular Ig-like domains of lipolysis-stimulated lipoprotein receptor (LSR) in the GI tract [77, 109, 111]. Once the CDTb is receptor-bound, it mediates the association of LSRs to form a lipid raft, followed by CDTa binding to the N-terminus of CDTb and subsequent internalization. The exact mechanism of endocytosis is unknown, however it is favoured towards a clathrin-independent pathway with dynamin activity [98]. Similar to TcdA and TcdB, endosome acidification is required for pore formation and translocation of CDTa to the cytosol, mediated by host cell factors Cyclophilin A and HSP70 [99, 101]. CDTa (48 kDa) targets the Arg-177 residue of G-actins (capping-like activity) and truncates the F-actin formation [102, 112], leading to G-actin/F-actin imbalance, cytoskeleton remodelling and subsequently cell death [113]. Interestingly, CDT can induce the formation of up to 150 µm long microtubule-based cell protrusions, forming a dense network on the cell surface, which is thought to assist in bacterial adherence [105, 114, 115]. Furthermore, fibronectin, a *C. difficile* receptor [116, 117] found within Rab11-positive vesicles of epithelial cells, are misdirected to the apical membrane of epithelial cells via the microtubules due to actin depolymerization [114, 115]. Thus, CDT has a key role in host-cell modulation during infection.

## Extracellular colonization factors

### Flagella

Flagella are commonly responsible for bacterial motility, evasion of host defences and colonization of apical surfaces. Within *C. difficile *, various ribotypes are differently flagellated; ribotype 012 strain CD630 is peritrichously (multiple flagella all over bacterial surface) flagellated, whilst the ‘hypervirulent’ ribotype 027 R20291 possesses a single monotrichous flagellum [118].

As seen in other bacteria, *C. difficile* flagellum has three distinctive components, the membrane bound basal body, hook and a helicoidal filament encoded on three operons termed F1, F2 and F3 [119, 120]. The F3 operon encodes for FliA, which is thought to regulate the late genes of F3 [120]. F2 is the inter-flagellar region for glycan genes, which varies across ribotypes. The glycosylation of flagella confers increased stability, enables cell–cell interactions and elicit host responses [121–123]. Transcription of F1 genes, which encode the flagellin (FliC), cap (FliD) and post-translational modification of the flagellar filament, occurs after F3 protein assembly [118–120, 124]. The flagellar alternative sigma factor, SigD, encoded on F3 is activated and mediates the transcription of the late-stage operon [125, 126]. SigD is also regulated by the orientation of a 154 bp invertible sequence, termed the flagellar switch, upstream of the F3 regulon [124, 127].

In *C. difficile,* the role of the flagella has been associated with colonization and virulence in the hamster and murine infection models [118, 125, 128], although there is strain specificity in the phenotypes associated with flagella. Flagellar mutants in CD630 were able to adhere at a greater capacity to Caco-2 cells, when compared to the parental strain. Conversely, R20291 exhibited a decreased ability in identical conditions [118, 128]. Furthermore, a higher toxicity was recorded in CD630 flagellar mutants when exposed to Vero cells and in a hamster infection model, when compared to the WT strain [128]. Mutations in the F1 operon yielded an increase in toxin production, although a reduction in toxin production was observed in isogenic mutants of the F3 operon, indicating possible regulatory roles of certain flagellar genes (*sigD, fliC* and *fliD*) in overall bacterial virulence [125, 126, 128, 129]. Therefore, the flagellum is clearly important in *C. difficile* infection, although its precise role, which appears to be strain-dependent, remains elusive.

### Type IV pili

Pili structures in *C. difficile* were first described by Borriello *et al.,* over 3 decades ago as fimbriae, describing multipolar protrusions of approximately 4–9 nm diameter and 6 µm long, although no correlation between binding efficiencies of strains with/without these fimbriae were demonstrated [130]. The *C. difficile* genome encodes the type IV pili (T4P) system, consisting of nine different pilin genes, assembly and scaffold proteins [131, 132]. The most studied genes are *pilA1* and *pilB1*, which encode for the major pilin and pilus assembly ATPase, respectively [131–133]. Bacterial virulence associated with T4P has been attributed to increased biofilm formation, direct adhesion and colonization to epithelial cells [133–135].

Pilus-like filaments were demonstrated using immunogold labelling with PilA antisera in *C. difficile* strain 630 adhering to crypt cells of hamsters [136]. Mutants of *pilA1* or *pilB1* in CD630 exhibited a reduction in adherence to Caco-2, HT-29 epithelial cells and MDCK cells. The adherence defects were more pronounced in *pilB1* mutants, indicating that the assembly of minor pilins play a functional role in adhesion and persistence [135]. Deficiencies in auto-aggregation and early biofilm formation were also demonstrated in *pilA1* mutants *in vitro* using confocal microscopy [131]. Furthermore, an analysis of pilin gene expression in R20291 and CD630 from planktonic and biofilm phases, showed that *pilA1* was upregulated threefold in R20291 suggesting a T4P-mediated biofilm formation in this strain. Pilus-assisted motility was also higher in R20291 across agar concentrations compared to CD630 [133]. Interestingly, the downregulation of flagella associated with the increase in adhesion [118, 125] have been attributed to the T4P, the theory being that the flagella sterically hinders the ability for the T4P and other adhesins to bind [135].

### Surface layer proteins

*C. difficile* has an surface layer (S-layer) that is linked to the peptidoglycan-containing layer and accounts for approximately 15 % of the total protein within this bacterium. *C. difficile* is unique as this bacterium is coated by a mostly heterodimeric, proteinaceous paracrystalline array [137–141], which is composed of slightly varying glycoproteins; low molecular weight S-layer protein (LMW SLP) and high molecular weight S-layer protein (HMW SLP). The *C. difficile* S-layer shows a considerable degree of variation between strains and thirteen S-layer cassette types have been identified [142].

Strains are divided into two groups based on varying sizes of the glycoprotein: group I have 32 kDa LMW SLP and 42–48 kDa HMW SLP, while group II has 38 kDa LMW SLP and 42 kDa HMW SLP [143]. The HMW and MLW subunits are encoded by *slpA*. SlpA has three sub-domains: N-terminal secretion signal, LMW SLP and HMW SLP. The secretion signal translocates the peptide across the cell membrane by an accessory Sec system [9, 138, 140]. Post-translational cleavage occurs with a paralog of *slpA* called cell-wall protein 84 (cwp84) [9, 141, 143]. LMW SLP is highly variable among isolates indicating a role as an antigenic determinant and evading immune recognition [139, 143, 144]. Binding in between the end of LMW SLP and the peptidoglycan-containing cell wall is the highly conserved HMW SLP, which exhibits amidase activity [137]. An atomic level model of *C. difficile* S-layer assembly, based on high-resolution crystal structure and electron microscopy was recently reported, which surprisingly revealed a tightly packed array with narrow pores, possibly allowing the passage of small metabolites. While more flexible and permeable points in this compact structure are indicated, it remains to be understood how large proteins are secreted out through this layer [142].

The S-layer is thought to have a role in adhesion to cell surfaces and colonization. Utilizing purified recombinant SLPs, SLP was demonstrated to bind via the HMW SLP to collagen I, thrombospondin and vitronectin. High levels of avidity were also observed between LMW SLP to epithelial cell Caco-2 but paradoxically to Hep-2 cells [145]. Specific protease inhibitors [146], chemical removal of the S-layer [145], anti-SLP antibodies [9, 147] and transposon insertion [148] were previously used to inhibit the S-layer function, as the S-layer is considered essential for bacterial survival. A S-layer null mutant, which had pleiotropic effects including decreased toxin production, was shown to be avirulent in a hamster infection model, although the mutant was still able to colonize the gut. In addition, two strains of *C. difficile* have been isolated without a S-layer, accompanied with a reported reduction in spore production and survival rate, toxin release and increased susceptibility to lysozymes [149].

Furthermore, most bacterial S-layers are glycosylated, however, although *C. difficile* has the relevant gene clusters, there is a lack of evidence for glycosylation [140, 144]. There are approximately 30 other proteins within this paracrystalline array [138], which may suggest that the S-layer is not primary adhesin, but might act as another alternative high avidity component in the event of downregulation of other adhesin [147]. The essentiality of the S-layer dictates that the S-layer is important to the bacterial survival [146, 149].

### Cell-wall proteins

To date, 28 other cell-wall proteins (Cwp) have been identified in *C. difficile,* which are part of the S-layer. *SlpA* and 11 slpA paralogs can be found in the *slpA* locus and 17 more within the genome [138]. These paralogs encode for a N-terminal signal peptide alongside three putative cell-wall-binding domains and a variable domain, which gives the Cwp its distinctive feature and function [137, 141, 150]. The structural components of many Cwps have been determined however their functions have not been fully elucidated. A few Cwps including Cwp84, Cwp66, CwpV, SlpA, Cwp22 and Cwp2, have been studied for their role in attachment to cell surfaces.

Cwp84 mediates the cleavage of SlpA. As isogenic *cwp84* mutant generated uncleaved SlpA peptides, which would translocate onto the cell surface; these mutants grew slowly and had a propensity for aggregation [151, 152]. However, the capacity for virulence was unaffected in a hamster infection model, suggesting Cwp84 may not be an essential virulence factor [151]. Additionally, Cwp84 is able to degrade extracellular matrix molecules fibronectin, vitronectin and laminin [153].

Cwp66 was one of the first proteins to be associated with cell adhesion, conferring an increased adhesive ability to Vero cells, which was reversed upon treatment with anti-cwp66 C-terminus antibodies [154]. CwpV is phase variable and well conserved across all strains [152, 155], occurring in 0.1–10 % of total cells and comprising up to 13 % of the S-layer [156]. Mutants overexpressing CwpV exhibited small, clustered colonies with a decreased susceptibility to bacteriophage infections, however its role in adhesion is not clear [156, 157]. Cwp2 mutants of *C. difficile* 630 demonstrated decreased adherence to Caco-2 cells and increased toxin A release [158]. R20291 *cwp22* mutants on the other hand showed reduced toxin production, autolysis, cell growth and adherence to HCT-8 cells. Furthermore, a reduction in virulence and bacterial fitness was observed in a mouse infection model [159]. Cwp19 is another recently described peptidoglycanase with a lytic transglycosylase activity, with mutants exhibiting delayed autolysis and subsequently toxin release. Interestingly, this phenotype was only observed in a glucose-based media [160]. Thus, Cwp proteins appear to have various functions, with an important role in bacterial attachment, although the functions of many other Cwp proteins remain unknown.

### Other surface-associated proteins

Other surface proteins have also been associated with cell adhesion and virulence. Collagen binding protein A (CbpA, CD3145) was identified through an *in vivo* transcriptomics study of *C. difficile* 630 murine infection. CbpA displayed an increased expression rate early in infection and was shown to interact with collagen and adhere to extracellular matrix producing cells [161, 162]. Tulli *et al.* demonstrated a potential redundancy in proteins binding to collagen, which may explain its lack of impact on bacterial colonization *in vivo*. CD2831, another collagen binding protein was also reported to bind to collagen-rich tissues such as human fibroblasts [163]. The surface protein Fbp68 was shown to bind to fibronectin and Vero cells, and antibodies raised against Fbp68 were able to partially inhibit binding [116]. However, mutants of Fbp68 adhered more to Caco-2 and HT-29 epithelial cells, further suggesting redundancy or compensatory control of adhesins. *In vivo* infection in monoxenic mouse, *fbp68* mutants had a reduced capacity to colonize the caecum, while in dixenic mice the colonization rates were similar, albeit slightly slower [164].

A lipoprotein CD0873 was discovered to be involved in adhesion to Caco-2 cells, with a mutant lacking this protein displaying a reduction in attachment and antibodies against CD0873 inhibiting adherence [165]. Using a competition assay in dixenic mice, strains expressing this lipoprotein was able to outcompete the mutant strain. Immunization of these mice with recombinant CD0873 elicited a strong immune response preventing long-term colonization and persistence [166].

Finally, there are other secreted proteins that modulate bacterial adherence. A secreted zinc metalloprotease, proline-proline endopeptidase (PPEP-1), was reported to efficiently cleave CD2831 and CD3246 to mediate better adherence. Furthermore, PPEP-1 is able to cleave fibrinogen *in vitro* and fibronectin produced by human fibroblasts, indicating potential roles of extracellular metalloproteases in infection [167, 168].

Although functions of *C. difficile* surface proteins have been studied, further understanding of their role during infection is necessary, as these may have excellent potential as vaccine candidates that could help prevent and disrupt the process of *C. difficile* colonization. It is important to note that some of the putative multifunctional adhesins discussed above were characterized only using *in vitro* assays. The presence of multiple surface proteins in *C. difficile* makes it difficult to study specific functions during infection, however, this could suggest redundancy or specializations specific to the host environment. Additionally, challenges associated with genetic manipulation of *C. difficile* have limited targeted structure-function studies of surface proteins.

### Hydrolytic enzymes

Clostridia can express and secrete hydrolytic enzymes to increase their range of nutritional sources [169]. Enzymatic abilities attributed to *C. difficile* and other anaerobes from clinical samples were investigated, and were found to be able to produce hyaluronidases, chondroitin sulfatases, gelatinases, collagenases, proteases and heparinases [170, 171]. *C. difficile* may utilize hyaluronidase to degrade hyaluronate found as a major component of ground substance, a viscous substance secreted by connective tissues, which acts as a natural physical barrier to prevent bacterial penetration. The breakdown of this barrier may allow an easier course of infection for *C. difficile *. Furthermore, the substrates produced are dissacharrides, which are thought to provide another nutritional source for the bacterium [171, 172]. A similar mechanism is thought to exist for chondritin sulfatase, as chondritin sulphate are another glycosaminoglycans found on proteoglycans in the extracellular matrix and cell surfaces [173, 174].

## Mechanisms of bacterial persistence

*C. difficile* can persist in different environments including the human gut through two main mechanisms, formation of spores and biofilms.

### Sporulation

The anaerobic nature of *C. difficile* necessitates the need for stress-resistant spores to maintain its lifecycle outside of host environments. Initiation of sporulation is a common path utilized by *Bacillus* and *Clostridioides* spp. when exposed to environmental stresses such as nutrient limitations, quorum sensing, oxygen, desiccation and ultraviolet light [15, 175].

Sporulation frequencies vary between strains and in different laboratory conditions, with CD630 being the most studied *C. difficile* strain [50]. Like *Bacillus,* Spo0A acts as the master regulator for sporulation [176–178]. Transcription of *spo0A* coordinates expression and activation of sporulation sigma factors: σ^E^, σ^F^, σ^G^, and σ^K^ [177, 179, 180] and a negative regulator sinR [178]. The sporulation sigma factors engage at various stages and locations of spore development, σ^F^ and σ^G^ are active in the forespore, while σ^E^ and σ^K^ in the mother cell. Briefly, early sigma factors σ^F^ and σ^E^ are activated during the polar septum formation and mother cell engulfment, respectively. σ^F^ proteolytically activates late sporulation σ^G^, but unlike *B. subtilis *, lacks proteolytic activation of σ^K^ by the products of σ^G^. σ^K^ becomes active upon synthesis to contribute to the remainder of the sporulation pathway of cortex synthesis, exosporium assembly and release. The sequential order activation is not as completely clear in *C. difficile* and in general sporulation pathways in *C. difficile* are distinct to *Bacillus* [50, 177, 180].

While Spo0A also regulates biofilm formation, flagella production, expression of cell-wall proteins [178] and indirect regulation with the sin locus [176, 181, 182], modulators like RstA control sporulation and toxin production through the phosphorylation of Spo0A [183]. Three of five orphan kinases (PtpA, PtpB and PtpC) have been reported to negatively regulate sporulation [184, 185]. Both carbon catabolite protein A (CcpA) and CodY are global regulators associated with metabolic activity and repress sporulation. In response to sugars, CcpA binds to promoters of Spo0A and σ^F^ [186], while CodY is a negative regulator the *sinRR’* and *tcd* operon [187, 188].

The ubiquitous nature of *C. difficile* spores provides potential continual sources of infection, in both the nosocomial and community environments [189]. The ability to sporulate is associated with virulence and transmission of CDI; *spo0A* mutants of *strains* 630 and R20291 were able to cause acute disease but unable to persist or transmit infection in a murine infection model [190]. In a different study, post-vancomycin treated-mice infected with the parental strain developed recurrent infection, whilst mutants did not demonstrate any relapse [176]. Notably, strains exhibiting high levels of sporulation were associated with an increased chance of recurrent infections [191, 192], though paradoxically under minimal growth conditions, hypervirulent isolates exhibited lower levels of germination compared to less virulent isolates [193]. Spores can also subvert the host immune system [194], induce cytotoxicity [195], adhere to intestinal epithelial cells [196–198] A recent study reported that spores internalized into gut epithelial cells could be another way in which spores contribute to recurrence of CDI [196].

### Biofilm formation

Biofilm formation is believed to be involved with the progression of colonization, maintaining persistence and resisting environmental stresses, with a positive correlation of the duration of hospital occupancy and biofilm-associated infections with other species [199–201]. Therefore, *C. difficile* biofilms may contribute to prolonged and repeated episodes of infection [202, 203].

*C. difficile* can form either mono- or poly-species biofilms with other gut bacteria [200, 204–206] both *in vitro* and *in vivo* [204–207], influenced by a plethora of factors such as the pili, flagella, cell surface components, quorum sensing and sporulation [131, 133, 200, 206, 208–210]. External factors such as exposure to sub-inhibitory concentrations of bile salts, prebiotics and antimicrobials, metronidazole and vancomycin can induce biofilm formation [58, 211–213], which in turn, increases the antibiotic tolerance [214]. The dense polysaccharide biofilm matrix can act as a physical barrier to resist antimicrobial penetration, increase oxygen tolerance and hold intra-matrix antimicrobial degrading enzymes [214–217]. Additionally, certain strains of *C. difficile* biofilm can harbour dormant spores with reduced germination efficiency [208].

*In vivo C. difficile* is primarily associated with the caecum and colon [205, 207]. *In vivo* studies in murine models suggests that formation of biofilms occurs on the outer layer of the mucosal lining of these epithelial cells and in conjunction with other *Bacteriodota* and *Bacillota* [205, 207]. Furthermore, biofilms harbouring *C. difficile* were shown to cause recurrent infection in a human gut model [202]. While the contribution of biofilms in persistence and colonization cannot be overlooked, the variability in biofilm regulation and formation between strains poses a challenge for researchers [218].

## Host interactions of *C. difficile*

Most work at the epithelial interface has been performed with the toxins A, B and CDT. Multiple cellular receptors have been reported to interact with toxin A and toxin B. In a recent CRISPR-Cas screen, sulfated glycosaminoglycans and low-density lipoprotein receptor were identified to impact toxin A endocytosis [219]. Toxin B interacts with the Wnt signalling Frizzled proteins and the chondroitin sulphate proteoglycan 4, structural details of these interactions have been characterized [220, 221]. As described earlier, CDT receptor binding, endocytosis and impact on host actins has been well studied. Endocytosis of toxins result in actin skeleton reorganization, and ultimately cell rounding and death. Host cells also respond to toxins by increasing levels of innate mechanisms like hypoxia inducible factor 1α, which regulates protective factors like vascular endothelial growth factor A, CD73 and intestinal trefoil factor, which are important for anti-apoptotic or epithelial repair processes [222]. However, repair of epithelial cell damage mediated by *C. difficile* infection was also shown to be impacted by stem-cell damage induced by toxin B [223].

A few other bacterial factors have been described to utilize host pathways. Heme transporter system encoded by *C. difficile hsmRA* was shown to capture and utilize haem, thus reducing the inflammatory response it induces, while another haem system HatRM system detoxifies haem through efflux [224, 225]. Furthermore, ZupT, a metal import system was reported to compete against calprotectin-mediated growth inhibition [226]. Competition assays in mice revealed a defect in colonization in a *zupT* mutant, suggesting metal homeostasis is key for infection [226]. Furthermore, calprotectin, a zinc binding protein, which impacts *C. difficile* growth by limiting Zn availability, was shown to be an important factor in protection against CDI [227].

Finally, the mucus is a key host component that gut pathogens need to interact with. Engevik *et al.* reported decreased MUC2 production in CDI patients, with no changes in MUC1 secretion [228]. They also suggested increased binding of *C. difficile* to stool mucus from patients with CDI. *C. difficile* was further shown to chemotax towards MUC2 and utilize mucin-derived oligosaccharides. However, as *C. difficile* lacks the glycosyl hydrolases required to degrade mucins, it is unable to utilize MUC2 directly, and breakdown of mucins to monosaccharides by other mucin degrading gut commensals was necessary [228, 229]. Furthermore, competition with similar bacterial mucosal sugar utilizers resulted in the decreased *C. difficile* colonization in mice [230].

## Immune responses to CDI

The innate immune response plays a key role in CDI, which is primarily been attributed to increased neutrophilic infiltration at the gut mucosa. Antibody responses against toxins have been well studied although recent work has highlighted epithelial immunity mechanisms in CDI. It is evident now that a plethora of complex interactions between both arms of immunity, the bacterium and the microbiota impact the disease severity and clinical manifestation.

### Innate immune responses

The classical symptom of pseudomembranous colitis in severe CDI is associated with epithelial tissue damage and heavy inflammation from neutrophil infiltration [231, 232]. The inflammatory response to CDI can be initiated through luminal toxin expression, spores or surface proteins, triggering the release of neutrophil chemoattractants IL-8, CXCL1, CCL2, CXCL2, CXCL5, IL-6, TNFα and IFN-γ [139, 195, 233–238]. Neutrophil recruitment is a double-edged sword, as it is beneficial for combating foreign pathogens, but excessive recruitment can also cause localized damage. Neutrophil recruitment has been shown to protect mice from weight loss and improve clinical scores [34], and reduction of neutrophil recruitment led to increased mortality rates in mice [239, 240]. The role of neutrophils is also reflected in clinical cases, as patients suffering from neutropenia were more susceptible to recurrent CDI [241]. Severe CDI have been indirectly associated with neutrophilia, leukocytosis and increase in pro-inflammatory responses [242, 243]. Protection against neutrophil-mediated damage has been studied with anti-CD18 antibody against neutrophil recruitment and study of IL-23 deficient infected mice [244–246].

The role of eosinophils remains unclear in CDI, however Cowardin *et al.,* showed hypervirulent strains expressing the binary toxin (CDT) was able to suppress host eosinophil response through activation of the Toll-like Receptor 2 (TLR2)-dependent pathway; subsequent adoptive transfer of TLR2-deficient eosinophils was able to restore survival rates of TLR2^-/-^ mice [247]. *C. difficile* can also elicit the activation of other TLRs. Flagellin is recognized by TLR-5 on epithelial cells, which induces the MAPK and NFκB cell signalling cascade, resulting in an inflammatory response and mucosal injury [248]. The secretion of proinflammatory cytokines can be further enhanced by the presence of toxin B [249, 250]. Mice infected with FliC mutants displayed reduced mucosal inflammation, however, this can also be attributed to the decrease in bacterial motility [248]. Additionally, toxins A and CDT can also activate other inflammatory cascades mediated by the TLR2/6, TLR2-CD14 and TLR9 signalling pathways [251–253].

Phagocytic activity in CDI has been understudied, despite the abundance of macrophages in the lamina propria [254]. Toxin exposure to macrophages induces a stellate morphology with rounding of the perikaryon and an altered or loss of phagocytic function [254–256]. Toxin treatment of macrophages is accompanied with release of several proinflammatory molecules such as TNF-α and IL-1β in rat peritoneal cavities [257], anti-toxin ability of Substance P (in concert with TNF-α) in the rat ileum loop model [258]. A number of cytokines, IL-6, IL-8 IL-10, IL-12p70, CCL3 and CXCL2 are known to be induced *in vitro* in THP-1 and J774A.1 macrophages [259, 260]. Interestingly, macrophages can aid *C. difficile* persistence; spores were seen to survive in Raw 264.7 macrophages for up to 72 h and subsequently induce macrophage death [194, 195].

Recent studies have focused on another group of innate cells, the innate lymphoid cells (ILCs), which are further sub-categorized into ILC1s, ILC2s and ILC3s based on their differential developmental transcription factors, cytokine expression and effector functions [261, 262]. Geiger *et al.*, demonstrated that NFil3^-/-^ mice, which lack NK cells and ILC1s [263, 264], were more susceptible to CDI when infected with *C. difficile* spores VPI 10463 [265]. Rag1^-/-^ (B- and T-lymphocytes deficient) and Rag-γc^-/-^ mice (ILC and B- and T-lymphocytes deficient) were assessed using a similar infection model, and Rag-γc^-/-^ mice developed more severe CDI compared to the WT and Rag1^-/-^ mice [266]. Furthermore, the adoptive transfer of CD90^+^/CD127^+^ ILCs into the Rag-γc^-/-^ mice, resulted in recovery from infection. The study also distinguished the role of IFN-γ (ILC1s) and IL-22 (from ILC3s) in more severe cases of CDI, as Rag1 IL-22^-/-^ mice with IFN-γ neutralized, showed a higher mortality rate [266]. The role of ILC2s in CDI was uncovered through the examination of IL-33, which increased ILC2s activation in mice exposed to R20291. This resulted in lower mortality rates and reduction in epithelial disruption, independent of either bacterial load or toxin expression. The gut microbiota was highlighted as a key factor in IL-33 regulation, with faecal microbiota transplantation restoring IL-33 expression that was depleted upon antibiotic treatment [267]. The association between IL-22 and host microbiota has been reported by Nagao-Kitamoto *et al.*, suggesting IL-22 is able to regulate host glycosylation, promoting the growth of *Phascolarctobacterium* spp. and additional nutritional competition for succinate [268]. Further studies are necessary to understand the mechanisms underlying microbiota-mediated control of these cytokines and ILCs in CDI.

### Adaptive immune responses

Large numbers of T cells and B memory cells can be found in the lamina propria in CDI [269]. The interaction between commensal bacteria and the adaptive immune system is thought to be able to induce an ‘intestinal homeostasis’, with production of basal levels of anti-inflammatory regulatory T cells (Treg) and suppression of proinflammatory T-helper 17 cells (Th17), which provides a beneficial equilibrium [270, 271]. Treg cells produce anti-inflammatory cytokine IL-10, while Th17 cells produce pro-inflammatory cytokines IL-6, IL-12, IL-10, IL-17A, IL-17F, IL-21, IL-22, IL-23, IFN-γ, TNF-α and GM-CSF for bacterial clearance and maintenance of the epithelial barrier [139, 272, 273]. Under antimicrobial-induced dysbiosis, disruption of the gut microbiota leads to a reduction in TGF-β release, resulting in a Treg:Th17 disequilibrium, causing inflammation of the GI tract and alteration of epithelial cell permeability [270]. This imbalance has been highlighted through the adoptive transfer for Th17 cells, which leads to more severe CDI in a dextran sulphate sodium murine colitis model [273]. CD4^+^ T cells have been attributed to the prevention of recurrent infections [274], although they could contribute to severe CDI when combined with non-steroidal anti-inflammatory drugs [275].

Colonic biopsies of CDI patients and immunohistological assays revealed a reduction in B lymphocytes and mucosal macrophages, with larger decreases in recurrent episodes [269]. The circulation of memory B cells is integral to maintaining resistance to CDI. Immunosenescence, which affected B cell function, and which is often associated with ageing, could explain prevalence of CDI in the elderly [276, 277]. Maintenance of toxin B memory cell response was poor, as mice were unable to elicit protection upon rechallenging with *C. difficile* spores [278]. Sequencing revealed somatic hypermutations but limited isotype class switching in anti-toxin B antibodies in B memory cells and monoclonal antibodies generated displayed low to moderate affinity without a strong neutralizing ability [279]. Despite the brief longevity of circulating antibodies from B memory cells, anti-toxin antibodies have been long described to be effective at protecting the host against CDI. Anti-toxin A and B antibodies have been associated with protection against recurrent CDI [280–284]. While toxins have been central to vaccine strategies against *C. difficile *, vaccines based on toxins A and B have not yet been successful at phase-III clinicals trials indicating that other bacterial components may be needed for inducing effective protection.

## Conclusions

The interactions of the anaerobic pathogen *C. difficile* with the host and gut microbiota are highly intricate. A minor change in one dimension can alter *C. difficile* responses and the severity of the disease. Although significant advances have been made within the last two decades, there is a need for multifaceted assays to truly understand three-way interactions between these systems. However, such studies are challenging due the variability within systems, such as strain-dependent differences, diverse microbiota compositions and the lack of good model systems. Triangulating and understanding these dynamic processes will hopefully lead to new and better therapeutics for *C. difficile* infection.
